# Exploration of cfDNA landscape in NIPT and clinical utilities of cfDNA based gene expression inference in prenatal diagnostics

**DOI:** 10.3389/fgene.2025.1527884

**Published:** 2025-02-21

**Authors:** Ruo Jia, Jianjiang Zhu, Feng Zhang, Yangbo Sun, Bin Zhang, Yang Du, Hong Qi

**Affiliations:** ^1^ Department of Genetics, Shenyang Maternal and Child Health Hospital, Shenyang, Liaoning, China; ^2^ Prenatal Diagnosis Center, Beijing Haidian Maternal and Child Health Hospital, Beijing, China; ^3^ Department of Medical Genetics, Changzhou Maternity and Child Healthcare Hospital, Changzhou Medical Center, Nanjing Medical University, Changzhou, China; ^4^ Annoroad Gene Technology Co., Ltd., Beijing, China

**Keywords:** cfDNA, chromatin, fragmentomics, gene expression, hypothyroidism, NIPT, preeclampsia, WGS

## Abstract

Cell-free DNA (cfDNA) is a dynamic biomarker reflecting the physiological state of the body. Its unique physical and biochemical properties, inherited from the tissue of origin, enable a wide range of clinical applications. From methylation patterns and fragmentation profiles to genetic variants, cfDNA holds immense potential for diagnosing and monitoring various diseases, including cancer. In this study, we leverage a large collection of non-invasive prenatal testing (NIPT) dataset to explore the genomic landscape of fetal cfDNA, aiming to identify novel biomarkers associated with fetal development and maternal-fetal complications. Our study identifies novel fetal-specific genomic regions, further demonstrating the potential of cfDNA as a versatile biomarker. The prediction model achieved a 100% (12 of 12) positive predictive value (PPV) for hypothyroidism. Whereas for preeclampsia the PPV is much lower (25%, 3 of 12). By establishing a foundation for early hypothyroidism prediction and preeclampsia, we contribute to the expanding applications of NIPT. This approach can be adapted to explore other complex phenotypes and inform biomarker discovery, ultimately advancing maternal-fetal medicine.

## Introduction

Cell-free DNA (cfDNA) is DNA circulating freely in the bloodstream, unbound by cell nuclei. Released from cells through processes like apoptosis and necrosis, cfDNA levels fluctuate based on factors such as cell death rates, tissue origin, and clearance efficiency. A predominant feature of cfDNA is its nucleosome-sized fragments, approximately 166 base pairs long, resulting from DNase I-mediated cleavage (Jiang et al., 2020). The molecular characteristics of cfDNA, including fragment length, can vary depending on the tissue source (Shi et al., 2020).

Cell-free DNA is a valuable diagnostic tool due to its diverse origins and dynamic properties, reflecting the physiological state of the body. The presence of fetal cfDNA in maternal blood has enabled the development of non-invasive prenatal testing (Lo et al., 1997). Additionally, cfDNA derived from tumors or transplanted tissues serves as a sensitive biomarker for cancer monitoring and post-transplant surveillance (Agbor-Enoh et al., 2018; Wan et al., 2017; Lo et al., 1998). Even in healthy individuals, cfDNA can indicate viral or bacterial infections, expanding the potential applications of cfDNA-based diagnostics (Long et al., 2016; Tong et al., 2022).

In actively transcribed genes, the promoter region, about 150 bp upstream of the transcription start sites (TSS) is a nucleosome-depleted region (NDR) that facilitates access to the bulky transcriptional machinery and is flanked by arrays of well-positioned nucleosomes (Ulz et al., 2016). Its nucleosome-free promoter regions and characteristic fragmentation patterns offer insights into chromatin structure and transcriptional activity. Epigenetic modifications, such as DNA methylation, influence cfDNA cleavage patterns, with hypermethylated CpG sites exhibiting increased cleavage (Zhou et al., 2022). Fragmentomic markers including cfDNA fragment size and various types of fragment end motifs have been actively investigated, with substantial biological and clinical implications (Shi et al., 2020).

Cell-free DNA is a valuable biomarker reflecting the genomic landscape of its cellular origin. In this study, by analyzing the fragmentomic features of cfDNA from non-invasive prenatal testing samples, we aim to identify novel biomarkers associated with fetal development and maternal-fetal complications.

## Materials and methods

### DNA extraction and sequencing

Peripheral venous blood (5 mL) from each patient was preserved and delivered to the laboratory in EDTA tubes (Sekisui, Tokyo, Japan) or Streck tubes (La Vista, NE, US). Plasma was separated after 2 rounds of centrifugation and stored at −80°C until DNA extraction. Cell-free DNA was extracted from plasma according to standard commercial protocols, together with library preparation using China National Medical Products Administration (NMPA) approved kit (Registration No. 20173400331). Subsequently, 4.2 million single-end reads of 40 bp (SE40) were generated for each sample library using NextSeq 550AR (Annoroad Gene Tech, Beijing, China). All procedures were performed in a standard negative-pressure laboratory with constant temperature and humidity.

### Definition of fetus specific regions

Approximately 5,000 commercially tested NIPT samples from karyotypically normal male fetuses were retrospectively collected and stratified into 40 groups based on fetal fraction estimated using conventional method based on chromosome Y dosage. Each group contained approximately 125 samples with a median of 493.6 million reads (minimum 475 million) and a fetal fraction between 5% and 20%. Raw sequencing data of SE40 was initially aligned to the human reference genome (hg19) with BWA (Li and Durbin, 2009). Duplicated reads were marked using samtools (Li et al., 2009), and finally only uniquely mapped reads were kept. The uniquely mapped reads without duplication of each fetal fraction group were then merged into one concatenated BAM file, resulting in 40 merged libraries with raw reads number around 490 M.

Per-base depth of each merged libraries were first summarized. A regression model was built between per-base coverage and the average fetal fraction of each group. A commonly covered region on chromosome Y is called fetal specific if it falls around the fitted regression line with a fitted error less than 1.5%. Or in other words, the coverage of the fetal specific region changes linearly as the fetal fraction across groups. Consecutive fetal specific regions with at least 2 reads covered was taken and merged on chromosome Y. In a next step, we looked for covered regions on other chromosomes yet with similar level of correlation between fetal fraction and genome coverage. We then applied a 50 bp window flanking of such fetal specific regions and checked for the nucleotide’s distribution along the window, resulting in a position weight matrix of 4 rows of A/T/G/C and 100 columns for each position.

### Refinement and validation of fetal fraction estimation model

Leveraging these high confidence fetal-specific regions, it is feasible to develop a fetal fraction estimation model for individual NIPT libraries with substantially reduced sequencing depth. We again retrospectively select a much larger collection of 10,000 individual NIPT samples from karyotypically normal male fetuses. An n-dimensional feature vector Y was constructed, where each element *y*_n_ represents the count of uniquely mapped reads aligning to the n-th fetal-specific region identified previously.

$$\text{fcY}=\beta_{0}+\beta_{1}y_{1}+\cdots +\beta_{n}y_{n}$$

A jackknife resampling approach was employed to minimize the sum of squared errors in fetal fraction estimation. In each iteration, 5% of samples were randomly excluded from model fitting. The final set of parameter estimates { *β*_0,_ *β*_1,_ …, *β*_n_} was determined as the median of estimates from all 1,000 iterations. Model performance was evaluated using Pearson correlation on an independent validation cohort of 5,000 male NIPT samples with known fetal fractions.

### Multiple dimensional fitting

We further investigated cases previously misclassified as aneuploid due to elevated Z-scores. These false positives often arise from placental mosaicism, where a mixture of normal and aneuploid cells dilutes the aneuploidy signal. Unlike true aneuploidies, these cases exhibit inconsistent Z-score patterns across different fetal fraction estimation methods (sex chromosome, fetal-specific regions, and aneuploidy-related chromosome coverage). A combined Z-score, incorporating deviations from expected fetal fraction, was calculated to identify potential false positives. To establish a statistical threshold, a reference dataset including a large number of true positive and false positive cases should be used to determine a reference cutoff to flag outlying false positives. The combined Z-score can serve as an independent marker to identify potential placental mosaicism in samples initially flagged as positive using traditional Z-score-based aneuploidy detection.

### Genome-wide scanning of open chromatin structure and inference of differential fetus/placenta gene expression

Fetal fraction is an indicator of fetal development. Therefore, transcriptional activity of development related genes could be reflected in the open chromatin status of cfDNA. We further used the 20,000 per-base depth profiles collected earlier, to construct four new merged libraries with average group fetal fraction of 5%, 10%, 15% and 20% respectively. Each library contains equal number of 5,000 individual libraries. Per-base depth within each merged library was normalized by total read count. To reduce data dimensionality, an initial filtering step was applied to retain positions exhibiting consistent fold-change patterns across fetal fraction groups. This filter only included regions with monotonic relationship between per-base coverage and fetal fraction, regardless of the magnitude of the change.

By intersecting the previously identified fetal-specific regions with those exhibiting consistent fold-change patterns across fetal fraction groups, we defined a set of candidate regions indicative of potential open chromatin structures. A differential coverage analysis similar to RNA-seq methods (Love et al., 2014) was applied to these regions. Fold-change related test statistics were recorded and corrected for multiple testings. Regions demonstrating consistently significant fetal-specific enrichment were prioritized as potential markers of fetal development and associated phenotypes.

We then extended the single nucleotide candidate marker list by a 12 bp flanking in both directions, and evaluated the continuity of possible overlapping 25 bp windows, only focusing on those whose fold-change is negatively correlated with fetal fraction. Such continuous segments correspond to a more opened chromatin structure with increased transcriptional activity likely of fetus origin. The number of such continuous windows drastically decreases as expected. We then empirically cut the frequency screeplot by selecting those consecutive regions of at least 5 windows, which is illustrated in Figure 4A. Genes downstream of these regions were collected and subjected to a functional gene set enrichment analysis via DAVID (Huang et al., 2009).

### Cleavage profile of NIPT sample with clinically significant phenotypes

A retrospective study was conducted on NIPT samples from patients who subsequently developed early-onset preeclampsia. NIPT data of 80 sample libraries with confirmed early onset preeclampsia were pulled from data archive of 3 hospitals. The 80 individual NIPT sequencing data were merged and similarly processed as described earlier. Per-base coverage was calculated and visualized in pair with the 10% fetal fraction set data constructed in the previous section. The difference in the coverage profiles between preeclampsia and control groups were scanned across the whole genome, especially around the transcription start sites of known genes. When visualizing pair of coverage profiles on the gene level, the per base depth value was normalized again within the viewing area to further alleviate different numerical baselines between profiles.

Significancy of the differences were evaluated in order to derive a short list of loci with prediction potential. A subset of loci with pronounced differential coverage was selected for further analysis, using a similar setting as in the previous section when comparing between fetal fraction groups, in this case a binary contrast between preeclampsia and normal group. Using these loci windows as a whole, similar to a small synthetic chromosome, an analogous form of predictive score as NIPT Z-score can be built with a set of normal background samples. One particular consideration was taken into account of summing up read count mapped to regions showing opposite directions of between groups changes. A signed sum was used, for regions showing lowering coverage in the case group, the reads mapped to those regions was subtracted instead. As a result, higher scores indicated a greater likelihood of preeclampsia based on the identified coverage patterns. It is also possible to construct two sets of target statistics depending on their direction of change.

A retrospective analysis of 2,170 NIPT samples collected between August and December 2023 from Haidian Maternal and Child Health Hospital was conducted, covering all NIPT tested subjects within the period. A reference model was established using the entire dataset blindly, against which individual samples were compared to generate prediction scores. This is slightly different from constructing a typical NIPT reference dataset, in which case only known euploid samples were used. Inclusion of positive samples in the reference dataset might reduce sensitivity but would still preserve relative ranking of test samples. These scores were adjusted by adding a constant of 4, with negative values set to zero.

## Results

### Feto-placental specific regions and fetal fraction estimation model

**FIGURE 1:**
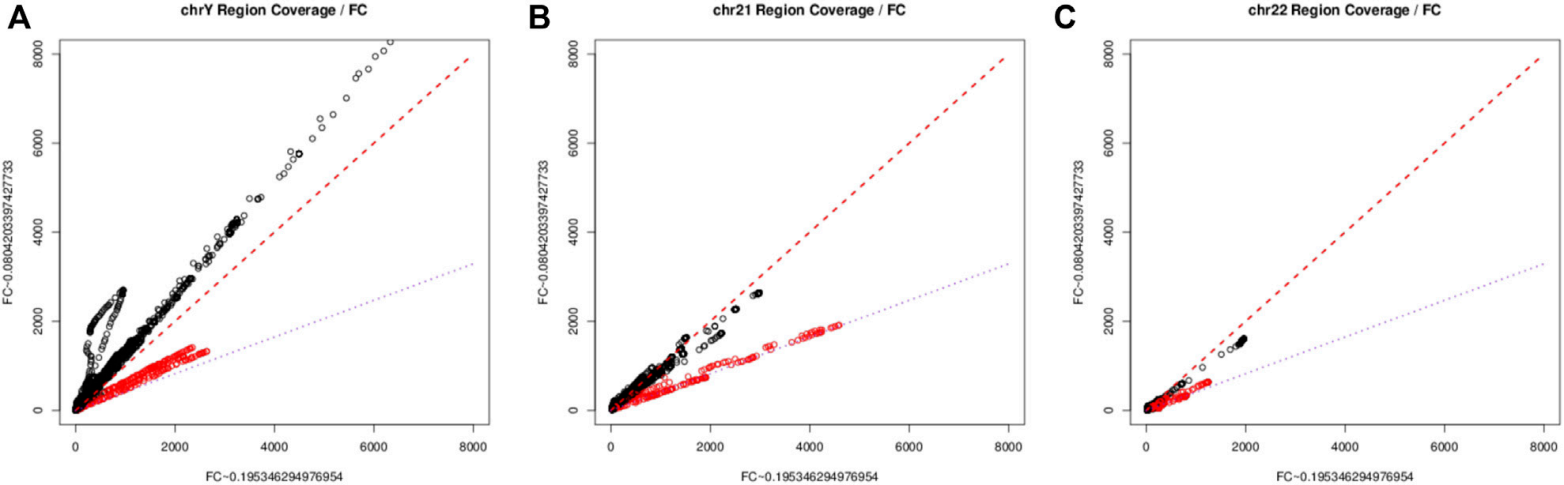
The scatter plots of depth of commonly covered regions between two fetal groups. chromosome Y [**(A)**, left], chromosome 21 [**(B)**, center], chromosome 22 [**(C)**, right). The red line represents the 45° identity line, while the purple line signifies the ratio of estimated average group fetal fraction. The minor deviation from the lines could be explain by the over dispersion resulted from the overly wide dynamic range in sequencing, together with the imperfect normalization method.

We explored such regions by first building a regression model between per-base coverage and the pooled estimated fetal fraction of each group, and found that across all regions only as subset of regions following the relationship of the fetal specific read depth and the fetal fraction, this is particularly true when we are inspecting the depth pattern on chromosome Y (Figure 1A). For those loci fall on the purple line, the change in depth is consistent with the between group ratio of average fetal fraction. This finding aligns with recent studies demonstrating the utility of specific sex chromosome regions for accurate fetal fraction estimation (Wang et al., 2016). On Chromosome 21 and 22, we applied a similar technique as on Chromosome Y, per-base coverage could be visually sorted into two groups (Figures 1B, C), suggesting a set of regions which follows the same trend as on chromosome Y and could be indicative for fetal fraction. The same approach has been applied on other chromosomes to identify fetal specific regions.

**FIGURE 2:**
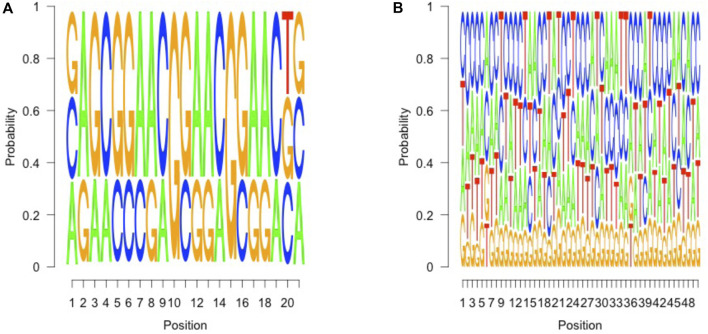
Sequence pattern of fetal specific regions. **(A)** (left), 6 to 26 bp in the 50 bp upstream of covered fetal specific regions, which suggests a marker pattern of AG CGGAACGGAA CG, which contains a short tandem repeat of CGGAA (3) units. **(B)** (right), in other covered regions, no such pattern could be identified in such 50 bp intervals.

**FIGURE 3:**
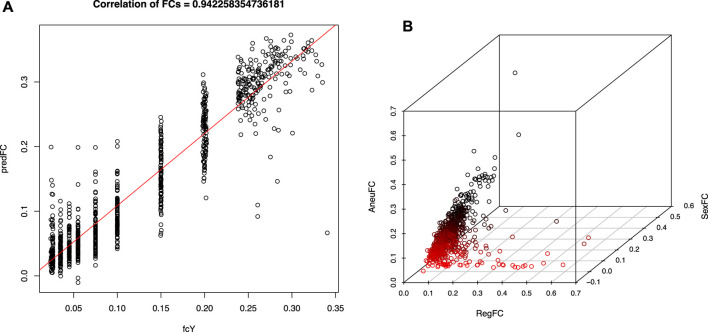
Fetal fraction and test statistics derived using fetal specific regions. **(A)** (left), Pearson’s correlation coefficient between fetal specific region fraction and traditional Y chromosome dosage estimates using validation dataset. **(B)** (right), previously predicted high risk samples of T13/T18/T21 trisomy illustrated in 3-D space expanded by aneuploidy signal (AneuFC), Y chromosome fraction (sexFC) and fetal specific region fraction (RegFC). A cluster of red dots were highlighted for those samples whose RegFC is significantly deviated from AneuFC when SexFC is not observed.

A high confidence sequence of nucleotides “CGGAA” could be derived from the flanking position weight matrix, suggestive of a potential transcription factor binding site, resembling a microsatellite structure, as depicted in Figure 2A. However, when we looked at the regions other than those fall along the purple line in Figure 1, no such pattern could be identified in the flanking intervals, as demonstrated in Figure 2B. Interestingly, it is known that transcription factors bind a CGGAA motif better when both cytosines in the CG dinucleotide are methylated (Yin et al., 2017; Ray et al., 2021). The refined fetal fraction model, leveraging genome-wide fetal-specific regions, demonstrated a strong correlation with the traditional Y-chromosome-based method, achieving a Pearson correlation coefficient of 0.9423 (Figure 3A).

A well-established relationship exists between NIPT Z-scores and fetal fraction in aneuploid samples, characterized by a linear correlation. Discrepancies between these metrics, particularly in cases with elevated fetal fraction and borderline Z-scores, may indicate potential false positives due to random sampling fluctuations or confined placental mosaicism (CPM). By incorporating region-specific fetal fraction estimates into an enhanced multidimensional fitting model, we identified previously misclassified aneuploidy cases (red dots in Figure 3B). These cases exhibited significant discrepancies between region-specific and overall fetal fraction estimates, suggesting potential false positives due to placental mosaicism. Given the screening nature of NIPT, cases identified as potential false positives through our refined model should still be managed as positive results in clinical practice to avoid missing true aneuploidies. Therefore, we did not further validate this model.

**FIGURE 4:**
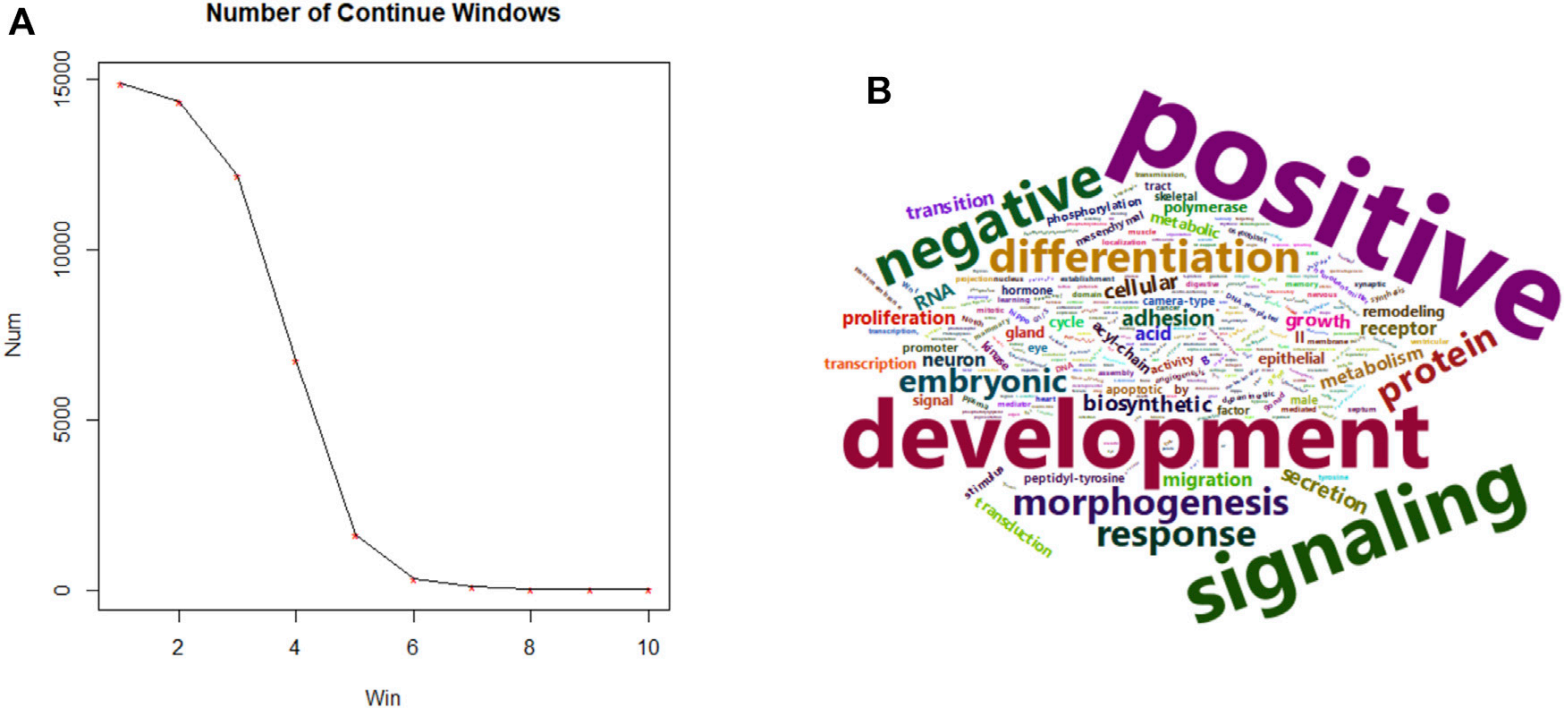
Size summary of fetal specific regions and transcriptional functions. **(A)** (left), size frequency of continuous 25 bp windows after intersecting fetal specific regions with windows carrying consistent lower coverage in higher fetal fraction groups. **(B)** (right), word cloud of top 30 GO terms using 1,657 predicted genes of higher fetal specific transcriptional activity.

Fetal fraction, as a proxy for fetal cell contribution, correlates with the transcriptional activity of developmentally relevant genes, which could be reflected in the open chromatin landscape of cfDNA. By intersecting fetal-specific regions with consistently underrepresented genomic segments across increasing fetal fraction groups, we identified 1,657 downstream genes. These regions, potentially associated with fetal regulatory elements, were enriched for gene ontology (GO) terms related to embryonic development, particularly in tissues such as the digestive tract, kidney, muscle, skeletal system, nervous system, and brain. These findings align with the expected temporal progression of fetal development after first trimester, within the NIPT sampling window. A word cloud emphasizing terms like “development,” “positive regulation,” and “signaling” (Figure 4B) further supports these observations. Detailed of the top 30 GO term enrichment results are presented in Table 1, with a minimum P-value less than 0.013.

**TABLE 1:** Top 30 enriched GO terms of gene set related to fetal specific regions.

Term	GeneNo	Overlap%	PValue
GO:0045893∼positive regulation of transcription, DNA-templated	65	4.11913815	3.77E-04
GO:0032148∼activation of protein kinase B activity	9	0.57034221	7.54E-04
GO:0048566∼embryonic digestive tract development	7	0.44359949	0.00109859
GO:0008284∼positive regulation of cell proliferation	58	3.67553866	0.00112482
GO:0042445∼hormone metabolic process	6	0.38022814	0.00168902
GO:0007517∼muscle organ development	17	1.07731305	0.00194273
GO:0007506∼gonadal mesoderm development	5	0.31685678	0.00229907
GO:0045669∼positive regulation of osteoblast differentiation	13	0.82382763	0.00276343
GO:0010595∼positive regulation of endothelial cell migration	11	0.69708492	0.0032291
GO:0001501∼skeletal system development	22	1.39416984	0.00334284
GO:0008584∼male gonad development	17	1.07731305	0.00344918
GO:0007507∼heart development	27	1.71102662	0.00348898
GO:0001837∼epithelial to mesenchymal transition	9	0.57034221	0.00490232
GO:0007283∼spermatogenesis	47	2.97845374	0.00501205
GO:0008285∼negative regulation of cell proliferation	48	3.0418251	0.00519815
GO:0007420∼brain development	27	1.71102662	0.00577131
GO:0010719∼negative regulation of epithelial to mesenchymal transition	7	0.44359949	0.00672
GO:0007338∼single fertilization	12	0.76045627	0.00710376
GO:0001525∼angiogenesis	30	1.90114068	0.0075737
GO:0007417∼central nervous system development	19	1.20405577	0.0078835
GO:0045786∼negative regulation of cell cycle	9	0.57034221	0.00839841
GO:1900042∼positive regulation of interleukin-2 secretion	4	0.25348542	0.00879714
GO:0001822∼kidney development	15	0.95057034	0.00892399
GO:0045944∼positive regulation of transcription from RNA polymerase II promoter	101	6.40050697	0.00935017
GO:0007399∼nervous system development	36	2.28136882	0.0097276
GO:0007169∼transmembrane receptor protein tyrosine kinase signaling pathway	16	1.0139417	0.01009885
GO:0007548∼sex differentiation	7	0.44359949	0.01054713
GO:0007219∼Notch signaling pathway	18	1.14068441	0.01109893
GO:0035115∼embryonic forelimb morphogenesis	8	0.50697085	0.01254616
GO:0002040∼sprouting angiogenesis	7	0.44359949	0.01294749

### Utilization of phenotypically associated coverage profile

**FIGURE 5:**
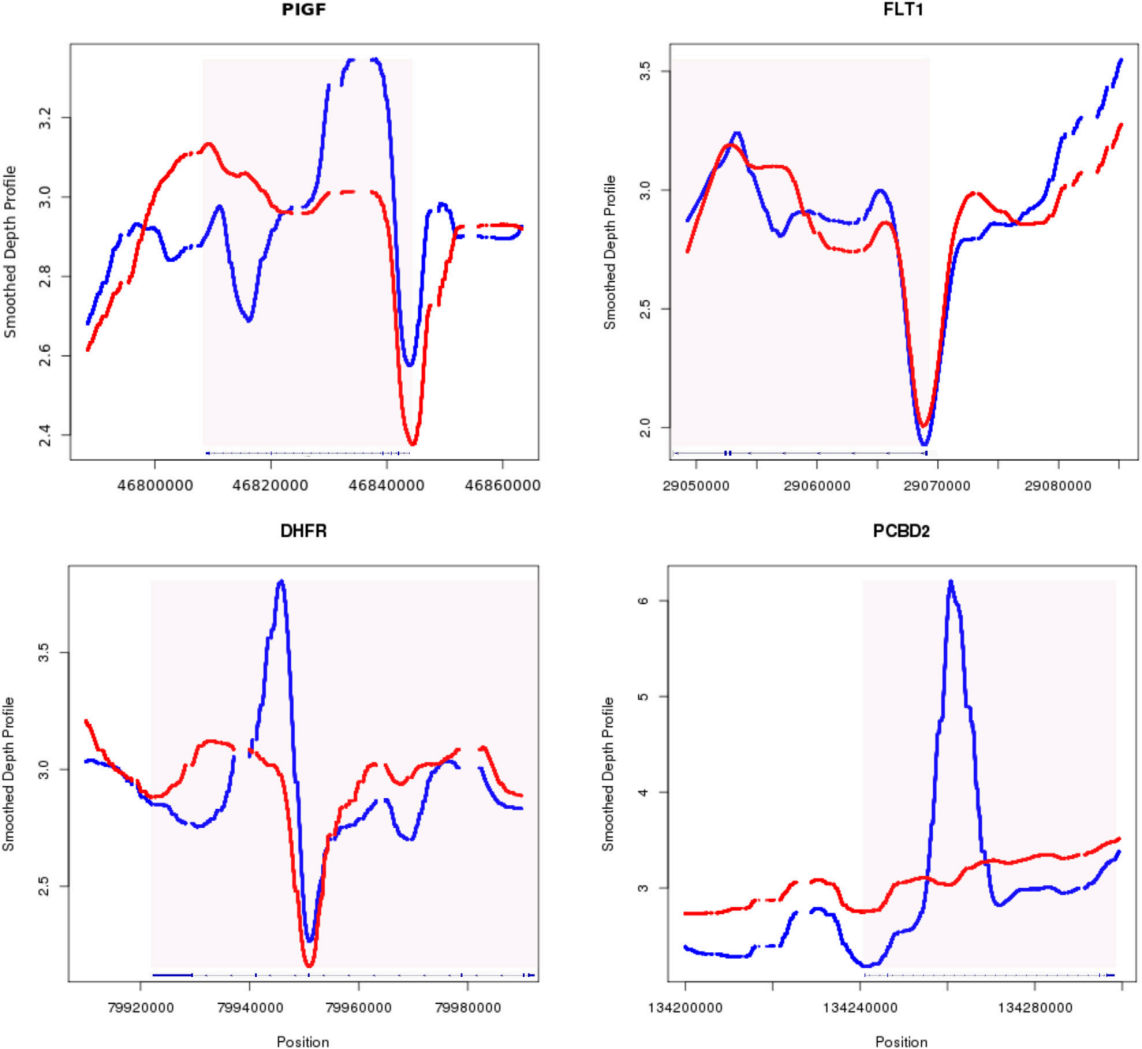
Smoothed depth profile of four example genes with potential clinical relevance of preeclampsia. PIGF (top left), FLT1 (top right), DHFR (bottom left), PCBD2 (bottom right) the red border signifies the outer most UTR of the gene, with patient data in blue and control data in red.

It was established previously that serum expression of PIGF shows significant decrease in women who later had preeclampsia, as early as 12 weeks of pregnancy; however serological marker sFlt-1 only starts to elevate in high-risk patient after 20 weeks (Levine et al., 2004). Our limited patient-control dataset revealed a distinctive S-shaped pattern upstream of the PIGF gene in the preeclampsia group (blue in Figure 5). This observation aligns with the hypothesis that open chromatin facilitates active transcription. The increased cfDNA coverage within the PIGF gene body and its upstream 5′ UTR region in preeclampsia cases suggests potential transcriptional suppression of PIGF. While the FLT1 gene exhibited less pronounced differences between groups, a subtle decrease in upstream coverage was observed in the preeclampsia cases compared to controls. This trend suggests a potential shift towards a more open chromatin configuration in preeclampsia, which may contribute to the later observed increase in FLT1 expression.

After between groups scanning across the whole genome, we also identified genes with significant changes in their gene body, depicted in the lower track of Figure 5. DHFR encodes protein able to converts dihydrofolate into tetrahydrofolate. Dihydrofolate reductase deficiency has been linked to megaloblastic anemia and severe neonatal neurologic disease, when DHFR is mutated and lost its function (Cario et al., 2011). Other study also used folate deficiency as a differential diagnosis indicator for severe preeclampsia (Sisman et al., 2019). Our finding of reduced function of DHFR in the preeclampsia group aligning with its established role in preeclampsia pathogenesis. PCBD2, a key enzyme in L-phenylalanine metabolism, exhibited decreased expression in preeclampsia cases. This aligns with previous findings of elevated phenylalanine levels in preeclamptic patients (Prameswari et al., 2022), suggesting a potential link between reduced PCBD2 activity and disease pathogenesis. The observed changes in chromatin accessibility, characterized by altered nucleosome occupancy, may contribute to the dysregulation of critical genes like PCBD2. In both exemplary cases of DHFR and PCBD2, the significant differences of cfDNA coverage were identified on the gene body. It has been shown that complex epigenetic interaction between methylation of DNA and histone modification exists, where hypermethylation can lead to increased histone deacetylation (Baylin, 2005), resulting in a more compact chromatin structure and potentially increased cfDNA release.

**FIGURE 6:**
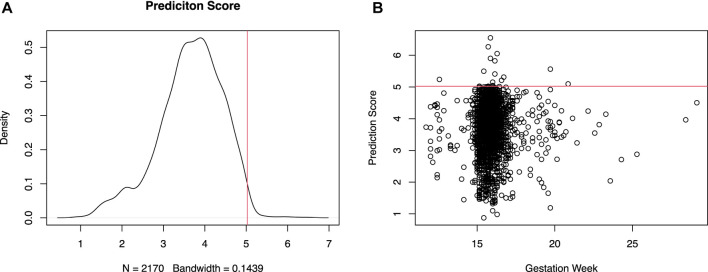
Distribution of test statistics for preeclampsia related disease prediction. Density of the prediction score [**(A)**, left] and scatter plot of prediction score versus gestation week [**(B)**, right] of a retrospective cohort.

With the constructed prediction model using peak features, we blindly tested 2,170 recent NIPT samples without any clinical information. 15 samples with prediction score above an empirical quantile of 99% (5.024214, in Figures 6A, B) were retrospectively checked after birth for any known clinical symptom identified during routine clinic visit along the gestation period. The 15 samples in the lower tail were also similarly reviewed. In Figure 6B, the prediction score shows no clear numerical relationship with gestational week. The 30 samples were all reported with low risk of aneuploidy in NIPT. None of the 15 patients in the lower tail have shown any sign of preeclampsia and other known clinically relevant phenotypes before or after the NIPT blood draw. Except for 3 (6.548891, 6.267465, 5.21113) of the high scoring patients who were later transferred to other hospital or lost contact, interestingly all other 12 of the 15 top scoring patients have been diagnosed with hypothyroidism, but without identified symptoms of preeclampsia at the time of NIPT sampling. 3 of the 12 have been later diagnosed with preeclampsia at gestation week of 37w (5.311052), 21w (5.237796) and 27w (5.596447) respectively. Technically, the model achieved a 100% (12 of 12) positive predictive value (PPV) for hypothyroidism. Whereas for preeclampsia the PPV is much lower (25%, 3 of 12). It is unclear whether hypothyroidism is an early contributing factor of later onset preeclampsia, or just an independent pathway which further complicates the preeclampsia related symptoms by interfering with the host’s endocrine system. Nonetheless evidence have been shown that preeclampsia patients exhibited hypothyroidism with increased level of serum thyroid‐stimulating hormone (TSH) (Hajifoghaha et al., 2022).

## Discussion

Cell-free DNA is a versatile biomarker with applications spanning multiple clinical domains. Methylation, fragmentation patterns, and genetic variants (single nucleotide variations, insertions, deletions, structural variants) serve as valuable biomarkers for various disease conditions including cancer (Vanderstichele et al., 2022; Han and Lo, 2021). cfDNA-based NIPT has become a cornerstone of prenatal care, with widespread adoption for detecting fetal aneuploidies. The technology’s potential extends beyond this, encompassing the screening of fetal subchromosomal copy number variations (CNVs) through low-pass whole genome sequencing or targeted approaches (Luo et al., 2019). Beyond aneuploidy detection, cfDNA analysis has expanded to encompass fetal monogenic disease screening through targeted sequencing (Brand et al., 2023). Additionally, clonal deconvolution techniques utilizing SNPs and other polymorphisms have been instrumental in refining fetal fraction prediction (Zhu et al., 2021) and informing tumor subclone analysis (Tarabichi et al., 2021), both critical for accurate test interpretation. Approach used in this study could be adapted in any cfDNA based molecular testing to infer epigenetic changes along with other DNA variants. By integrating epigenetic and fragmentomic analyses, this methodology holds promise for discovering novel biomarkers and advancing our understanding of complex biological processes. Such multi-omics approach has the potential to unlock new diagnostic and therapeutic opportunities within maternal-fetal medicine and beyond.

Preeclampsia is a severe pregnancy complication characterized by new-onset hypertension (Magee et al., 2022) and proteinuria after 20 weeks gestation, posing risks to both mother and fetus (Dimitriadis et al., 2023). While traditional diagnosis relies on clinical symptoms and serum biomarkers (Levine et al., 2004), the underlying pathophysiology remains complex. Despite targeting preeclampsia, which is normally diagnosed after 20w of gestation later than the typical time frame of NIPT sampling, our prediction model based on genomic region coverage profiles unexpectedly identified a cohort of samples primarily associated with hypothyroidism. These samples exhibited significantly higher prediction scores compared to the general population. Thyroid hormones are essential for regulating metabolism, growth, and development. Hypothyroidism, a condition characterized by insufficient thyroid hormone production, has been linked to an increased risk of preeclampsia. Meta-analyses consistently demonstrate a higher incidence of preeclampsia among women with hypothyroidism compared to those with normal thyroid function (Toloza et al., 2022; Männistö et al., 2013). The precise mechanisms linking hypothyroidism to preeclampsia remain elusive. However, potential contributors include impaired placental function, compromised oxygen and nutrient delivery, inflammatory processes, and vascular dysfunction, all of which can contribute to the development of hypertension and proteinuria characteristic of preeclampsia. The underlying biology suggests that there might be tissue and disease condition specific epigenetic changes in the studied genome which directly or indirectly affect the preference of nuclease cleavage (Zhou et al., 2023). Further research is necessary to elucidate the precise mechanisms linking hypothyroidism to these epigenetic alterations and their subsequent impact on preeclampsia pathogenesis. Our findings suggest that the developed NIPT-based prediction model could serve as an early diagnostic tool for hypothyroidism. Early intervention through dietary modifications may potentially mitigate the risk of preeclampsia in susceptible individuals. Recent study has shown potential of improving thyroid function with polyunsaturated fatty acids (PUFAs)-enriched diet (Li et al., 2024).

## Conclusion

By employing a comprehensive analysis of low-pass whole-genome sequencing (WGS) NIPT data, we identified novel fetal-specific genomic regions associated with key developmental processes and maternal-fetal phenotypes. These findings underscore the potential of cfDNA as a versatile biomarker for a range of clinical applications beyond traditional NIPT, including liquid biopsy of cancer and non-invasive screening of monogenic diseases. The identification of fetal-specific genomic regions offers valuable insights for optimizing primer and panel design in cfDNA-based assays, thereby enhancing diagnostic sensitivity and specificity through improved hybridization efficiency. Our study is the first to show clinical perspective of using NIPT data to predict hypothyroidism in early pregnancy, with potential to further differentiate preeclampsia, offering a potential avenue for improved maternal and fetal health.

## Data Availability

All sequencing data are managed by individual hospitals and Annoroad Gene Technology Co., Ltd., and are not available for share according to the Human Genetic Resources Administration of China (HGRAC). The partially processed data for the analysis have been provided as supplement (suppl1.wgs.geneBodyDiff.xlsx). Other form of data snapshots is available upon reasonable request made to YD.
